# Tylosis with oesophageal cancer: Diagnosis, management and molecular mechanisms

**DOI:** 10.1186/s13023-015-0346-2

**Published:** 2015-09-29

**Authors:** Anthony Ellis, Janet M. Risk, Thiviyani Maruthappu, David P. Kelsell

**Affiliations:** Department of Gastroenterology, Royal Liverpool University Hospital, Prescot Street, Liverpool, L7 8XP UK; Department of Molecular & Clinical Cancer Medicine, Institute of Translational Medicine, University of Liverpool, L69 3BX Liverpool, UK; Centre for Cell Biology and Cutaneous Research, The Blizard Institute, Barts and The London School of Medicine and Dentistry, Queen Mary University of London, London, E1 4AT UK

**Keywords:** Tylosis, Focal palmoplantar keratoderma, Oesophageal cancer, iRHOM2, RHBDF2, OSCC

## Abstract

Tylosis (hyperkeratosis palmaris et plantaris) is characterised by focal thickening of the skin of the hands and feet and is associated with a very high lifetime risk of developing squamous cell carcinoma of the oesophagus. This risk has been calculated to be 95 % at the age of 65 in one large family, however the frequency of the disorder in the general population is not known and is likely to be less than one in 1,000,000. Oesophageal lesions appear as small (2–5 mm), white, polyploid lesions dotted throughout the oesophagus and oral leukokeratosis has also been described. Although symptoms of oesophageal cancer can include dysphagia, odynophagia, anorexia and weight loss, there may be an absence of symptoms in early disease, highlighting the importance of endoscopic surveillance in these patients. Oesophageal cancer associated with tylosis usually presents in middle to late life (from mid-fifties onwards) and shows no earlier development than the sporadic form of the disease. Tylosis with oesophageal cancer is inherited as an autosomal dominant trait with complete penetrance of the cutaneous features, usually by 7 to 8 years of age but can present as late as puberty. Mutations in *RHBDF2* located on 17q25.1 have recently been found to be causative. A diagnosis of tylosis with oesophageal cancer is made on the basis of a positive family history, characteristic clinical features, including cutaneous and oesophageal lesions, and genetic analysis for mutations in *RHBDF2*. The key management goal is surveillance for early detection and treatment of oesophageal dysplasia. Surveillance includes annual gastroscopy with biopsy of any suspicious lesion together with quadratic biopsies from the upper, middle and lower oesophagus. This is coupled with dietary and lifestyle modification advice and symptom education. Symptomatic management of the palmoplantar keratoderma includes regular application of emollients, specialist footwear and early treatment of fissures and super-added infection, particularly tinea pedis. More specific treatment for the thick skin is available in the form of oral retinoids, which are very effective but commonly produce side effects, including nasal excoriation and bleeding, hypercholesterolaemia, and abnormal liver function tests. Genetic counselling can be offered to patients and family members once a family history has been established. The prognosis of tylosis with oesophageal cancer is difficult to determine due to the limited number of affected individuals. In the last 40 years of surveillance, five out of six cases of squamous oesophageal cancer in the Liverpool family were detected endoscopically and were surgically removed. Four of five patients had stage 1 disease at presentation and remain alive and well more than 8 years later. This suggests that the presence of a screening program improves prognosis for these patients.

## Introduction

This review summarizes current diagnostic, management and treatment practices for the rare genetic disorder tylosis with oesophageal cancer in the context of the current understanding of the molecular basis for this disease. This review is intended for a general audience with an interest in this rare form of familial cancer from a clinical or biomedical perspective. The disease has commonly been diagnosed following observation of the tylosis skin condition and a family history, but more recent advances in DNA based diagnosis has allowed a more objective approach. Finally, we discuss unresolved issues, highlighting the early stage of research into the molecular processes underpinning this disorder.

### Disease name/synonyms

Tylosis with esophageal cancer (TOC) (OMIM 148500)

Tylosis - oesophageal carcinoma

Palmoplantar hyperkeratosis-esophageal carcinoma syndrome (ORPHA2198)

Palmoplantar keratoderma with oesophageal cancer

Clarke-Howell-Evans-McConnell syndrome

Howell-Evans syndrome

Bennion-Patterson syndrome

Keratosis palmaris et plantaris with oesophageal cancer

Keratosis palmoplantaris-esophageal carcinoma syndrome

### Definition

Tylosis with oesophageal cancer is characterised by thickening of the skin of the hands and feet (focal, non-epidermolytic form of palmoplantar keratoderma) associated with a very high lifetime risk of developing squamous cell carcinoma of the oesophagus (OSCC).

## Epidemiology

Tylosis with oesophageal cancer was first described in two large Liverpool (UK) families [1], the larger of which was reviewed in 1994 [2]. At that stage, 345 family members had been identified, 89 of whom had been diagnosed with tylosis with 57 still alive. It has since been determined that the two Liverpool families are in fact distant kindreds [3]. Similar but smaller pedigrees have been reported from Germany [4] the United States [5] Finland [6] Spain [7] and Brazil [8]. The prevalence of the disorder in the general population is unknown, but is likely to be less than one in 1,000,000.

## Clinical description

**Fig. 1:**
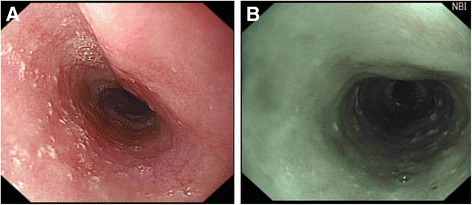
Benign oesophageal lesions in individuals from the Liverpool tyosis with oesophageal cancer family. **a** oesophageal lesions in a 32 year old female using conventional imaging; **b** oesophageal lesions in a 61 year old male using narrow band imaging

Tylosis (hyperkeratosis palmaris et plantaris) is a focal, non-epidermolytic form of keratoderma characterised by areas of yellowish thickened plaques restricted to areas of weight bearing and/or friction on the palms and soles [5, 9]. The cutaneous features are completely penetrant and are usually evident by 7 to 8 years of age but can present as late as puberty [2]. The skin lesions can be complicated by discomfort, fissuring and infection and may also include follicular papules and cutaneous horns. Oesophageal lesions present as small (2–5 mm), white, polyploid lesions dotted throughout the oesophagus (Fig. 1). The number and size of these varies between individuals, but does not worsen with age or prior to the development of carcinomas. In addition, oral leukokeratosis has been described, [10, 11]. Although the oral lesions are considered to be largely benign, two cases of squamous cancer of the oropharynx have been recorded (Ellis, personal observation).

A total of 21 out of 89 members of the Liverpool kindred reviewed in 1994 [2] had died of oesophageal neoplasia and 11 had died of other causes. The risk of developing oesophageal cancer in the Liverpool family was calculated to be 95 % at the age of 65. However, in this small sample size, the age of presentation in individuals with tylosis is not significantly lower than that for oesophageal squamous cell carcinoma (OSCC) in the general population (Ellis, personal communication), nor is distant metastasis more or less prevalent. There is no known increase in the incidence of other common carcinomas in these patients.

Symptoms of oesophageal cancer can include dysphagia, odynophagia, anorexia and weight loss. Initially the sensation of obstruction is only for solid food such as bread or meat and later it is also for softer food. In the later stages the patient has difficulty swallowing even liquids. The patient can lose their appetite or become afraid to eat (sitophobia) and they lose weight. If the site of the blockage is in the upper oesophagus the patient may choke or start coughing on swallowing due to inhalation of food and subsequently develop chest infections. Less commonly the condition may present due to spread elsewhere such as the lungs (breathlessness, cough), liver (jaundice, abdominal swelling due to fluid, ascites), bones (pain or unexpected fractures) or local lymph nodes (glandular swellings in the neck). However there may be an absence of symptoms, highlighting the importance of endoscopic surveillance in these patients.

### Aetiology

Tylosis with oesophageal cancer is inherited as an autosomal dominant trait with complete penetrance. Following linkage mapping and targeted next generation sequencing, missense mutations have been described in *RHBDF2* located on 17q25.1, which encodes an inactive rhomboid protein, iRhom2 [12], that plays a role in EGFR shedding. It is likely that aberrant EGFR signalling underlies the propensity for oesophageal carcinoma [13].

### Diagnosis and diagnostic methods

A diagnosis of tylosis with oesophageal cancer is made on the basis of a positive family history, characteristic clinical features, including focal palmar and plantar hyperkeratosis and oesophageal lesions, and mutations in *RHBDF2*. Oesophageal biopsies taken from individuals diagnosed with tylosis prior to the onset of oesophageal cancer do not have any specific distinctive features but tend to have prominent keratohyaline granules, inflammatory cell infiltrate and parakeratosis [14]. Histological features of the affected skin include acanthosis, hyperkeratosis and hypergranulosis but no parakeratosis or spongiosis. To date, three disease-associated missense mutations in *RHBDF2* have been identified:c.557 T → C (p.Ile186Thr), c.566C → T (p.Pro189Leu) and c.562 G → A (p.Asp188Asn) [6, 12].

Squamous cell oesophageal cancer associated with tylosis usually presents in middle to late life (from mid-fifties onwards) at a similar age to that of sporadic OSCC. The diagnosis is made by performing an oesophagogastroscopy (fibreoptic examination of the gullet and stomach) with biopsies taken of the tumour to establish the histological diagnosis and, if possible, the site within the oesophagus and the length of the tumour. The most common endoscopic appearance of the tumour is a visible swelling extending to a variable extent around the oesophageal wall and down the length of the oesophagus. It may be flat and plaque-like, or proliferative, reducing the lumen of the oesophagus. This is followed by a CT scan of the chest and abdomen to determine the presence of any local or distal spread.

### Differential diagnosis

As mentioned previously, oesophageal biopsies from individuals with tylosis do not have any specific distinctive features [14]. It is the combination of family history of both tylosis and oesophageal cancer, late onset skin lesions (i.e. substantially after birth but before puberty) and, more recently, the demonstration of mutation in *RHBDF2* that enable differential diagnosis [12]. However care should be taken to distinguish tylosis from other palmoplantar keratodermas (PPKs). The PPKs are a heterogeneous collection of skin disorders characterised by abnormal thickening of the skin of the volar surfaces of the hands and feet. They can be divided clinically into diffuse, focal and punctate types, histologically into epidermolytic and non-epidermolytic types and into hereditary and acquired types. Further subdivisions into simple (involvement of the palms and soles only), complex (involvement of non-volar skin including its appendages and, in certain cases, the buccal mucosa) and syndromic (associated with abnormalities in other bodily systems) are also made [15]. Diffuse epidermolytic PPK (EPPK) has been shown to be due to mutations in the gene encoding the palmoplantar specific keratin, *KRT9*, and in some cases *KRT1 *(16) whilst diffuse, non-epidermolytic PPK (NEPPK) has recently been associated with mutations in the gene encoding the water channel protein, Aquaporin 5 [17]. In focal PPK, the hyperkeratosis is less extensive and restricted to areas of weight bearing and/or friction. There are several types of focal PPK with abnormalities in other organs including hearing loss [18] and cardiomyopathy [19] due to defective gap junctions or desmosomes, respectively. Quite often the focal PPKs are complex and associated with abnormalities of the hair, nails, teeth and/or sweat glands particularly those associated with desmosomal mutations [19]. Using high throughput sequencing platforms, more genes underlying other forms of keratodermas are also being identified [20].

### Genetic counselling

Tylosis with oesophageal cancer is inherited as an autosomal dominant trait with complete penetrance. To date, three disease-associated missense mutations in *RHBDF2* have been identified: c.557 T → C (p.Ile186Thr), c.566C → T (p.Pro189Leu) and c.562 G → A (p.Asp188Asn) [6, 12] and form part of genetic counselling for the Liverpool family.

### Management including treatment

**Fig. 2:**
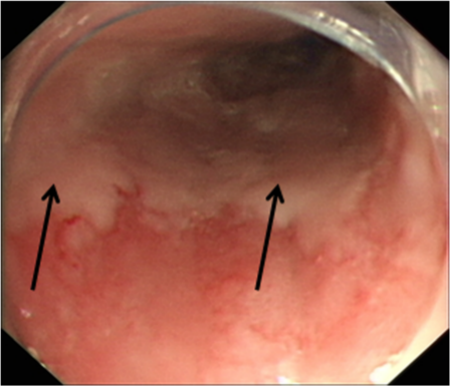
Dysplastic oesophageal lesion in tylosis with oesophageal cancer. Dysplastic area (arrowed) identified in a 57 year old male member of the Liverpool tylosis with oesophageal cancer family during routine screening

**Fig. 3:**
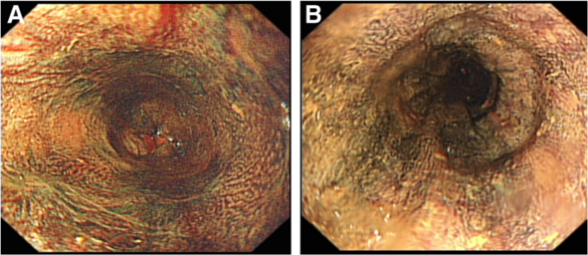
Chromoendoscopy. Oesophagus of **a** a 32 year old female; and **b** a 61 year old male in the Liverpool tylosis with oesophageal cancer family stained with 20 % iodine solution. Note that benign lesions are no longer discriminated from normal mucosa

The key management goal in patients with tylosis with oesophageal cancer is surveillance for early detection and treatment of oesophageal dysplasia. Since 1975, screening of members of the Liverpool family has included annual gastroscopy with biopsy of any suspicious lesion (Fig. 2) and quadratic biopsies (four biopsies equally spaced apart at any particular level) from the upper, middle and lower oesophagus to identify dysplasia. In the Liverpool family, it is recommended to start upper gastrointestinal (GI) endoscopy surveillance from the early twenties because one of the family members developed cancer at that age. In order to try and improve the sensitivity of the oesophageal surveillance, other endoscopic modalities have been tried, including narrow band imaging, but this did not result in earlier identification of precancerous lesions [21]. Further research is ongoing using chromoendoscopy (Smart HL, personal communication) during which a 20 % solution of iodine is sprayed via a cannula onto the wall of the oesophagus (Fig. 3). Areas of dysplasia and actual tumour show up as pale areas where the iodine dye has not been taken up by the oesophageal epithelium.

In addition to surveillance, all patients are given diet and lifestyle modification advice including smoking cessation and alcohol restriction, both of which are known risk factors for OSCC [22]. It is not known whether smoking and/or alcohol consumption are risk factors for oesophageal cancer development in individuals with tylosis because of the small number of patients, but it is likely that they are. Patients are encouraged to eat fresh fruit and vegetables [23] and are educated regarding the symptoms of oesophageal carcinoma such as dysphagia, odynophagia, anorexia and weight loss.

Symptomatic management of the tylosis includes regular application of emollients, specialist footwear and early treatment of fissures and super-added infection, particularly tinea pedis. More specific treatment for the thick skin is available in the form of oral retinoid tablets such as Etretinate and Acitretin. These treatments can be effective in causing the thick skin to regress but unfortunately side effects are common including nasal excoriation and bleeding, hyperlipidaemia, and abnormal liver function tests.

Sporadic oesophageal cancer usually presents in middle to late life (from mid-fifties onwards) as does the oesophageal cancer associated with tylosis, apart from the single exception mentioned above. If the tumour is deemed resectable then the treatment is surgery. If it is not resectable, then radiotherapy with or without chemotherapy is used. Local measures such as the insertion of a mesh stent may be employed depending on the size of the tumour to enable the patient to swallow while waiting treatment or if only palliative treatment is required.

## Prognosis

The prognosis of tylosis with oesophageal cancer is difficult to determine due to the limited number of affected individuals. Many of the oesophageal cancers in the Liverpool family occurred in earlier generations, however between 1975 and 2014, while the screening program has been operational, seven cases of squamous oesophageal cancer were reported. One of these patients had declined surveillance endoscopy and presented with an inoperable tumour (stage 4). Of the remaining six cases, five were detected during endoscopic screening while one presented with stage 3 disease between surveillance endoscopies, was surgically resected but died of disease. Four out of five cases were detected with stage 1 disease during screening and survived 8, 8, 8 and 29 years following resection, while the remaining tumour was detected as stage 3 disease with the patient dying of disease following resection.

Prognosis of OSCC depends greatly on stage with overall being poor (5-yr survival: <5 %) because at least 65 % of patients usually present with stage 2–4 disease which has already spread at least to regional lymph nodes. Our current data, albeit on only seven patients with cancer, suggests that the presence of a screening program improves prognosis for the Liverpool family members by detecting early stage disease. Patients with cancer restricted to the mucosa (stage 1) have about an 80 % survival rate, which drops to <50 % with submucosal involvement, 20 % with extension to the muscularis propria, 7 % with extension to adjacent structures (stage 3), and <3 % with distant metastases (stage 4).

### Unresolved questions

Key questions include those based around the role for iRhom2, encoded by *RHBDF2*, in keratinocyte and oesophageal biology. iRhom2 has been shown to regulate the trafficking and activation of ADAM17, a membrane bound sheddase which has been shown to play a pivotal role in the cleavage and release of membrane bound TNFα and EGFR ligands [24, 25]. Tylosis with oesophageal cancer is associated with gain-of-function mutations in the highly conserved cytoplasmic N-terminus of iRhom2 [6, 12]. As a consequence of increased ADAM17 activity, tylosis-derived keratinocytes show several features of “constitutive wound healing” in vitro, including rapid closure in scratch-wound assay, and upregulated shedding of ADAM17-dependent substrates such as EGFR-family ligands and TNFα [13]. EGFR pathway dysregulation has been implicated in several epithelial malignancies including sporadic squamous cell carcinoma of the oesophagus [26] and head and neck cancers [27]. It is likely that enhanced EGFR activity and TNFα shedding observed in tissue from patients with tylosis with oesophageal cancer facilitates tumourigenesis through aberrant and exaggerated wound healing, in response to stress within the oesophagus. This implies that lifestyle alterations to reduce physical or chemical damage to the oesophagus epithelium may lead to a reduction in cancer incidence in these individuals, although chemoprevention is also a clinical goal.

## Conclusions

Tylosis with oesophageal cancer is a rare, familial form of squamous cell oesophageal cancer that does not demonstrate early presentation. Identification of affected family members and initiation of a surveillance program for early detection may improve prognosis in these individuals. The identification of a causative gene allows the introduction of genetic screening into the diagnostic arena and paves the way for functional studies with the possibility of preventative therapies.

## Abbreviations

OSCC: Oesophageal squamous cell carcinoma
PPK: Almoplantar keratoderma
KRT: Keratin
